# Rab11 regulates E-cadherin expression and induces cell transformation in colorectal carcinoma

**DOI:** 10.1186/1471-2407-14-587

**Published:** 2014-08-12

**Authors:** Yuan-Chiang Chung, Wan-Chen Wei, Shin-Han Huang, Chi-Min Shih, Chih-Ping Hsu, King-Jen Chang, Wei-Ting Chao

**Affiliations:** Department of Surgery, Cheng-Ching General Hospital, Chung-Kang Branch, Taichung, Taiwan; Department of Life Science, Tunghai University, 1727, Sec.4, Taiwan Boulevard, Taichung, Taiwan; Department of Medical Laboratory Science and Biotechnology, Yuanpei University, Hsinchu City, Taiwan

**Keywords:** Rab11, E-cadherin, Colorectal carcinoma, Vesicle recycling, Epithelial mesenchymal transition

## Abstract

**Background:**

In the process of epithelial mesenchymal transition EMT, the disassembly of junctional adhesion complexes such as E-cadherin is a remarkable sign during changes in cell morphology and polarity. However, E-cadherin expression is dynamic, and is regulated by the cellular endocytic system; it is also involved in cell signaling mechanisms. In this study, we investigated the role of E-cadherin in colorectal tumors and the relationship with recycling endosome protein Rab11 in colon cell transformation.

**Methods:**

For tissue screening, the expressions of E-cadherin and Rab11 in colorectal tumors were identified by immunohistochemistry in 113 patients with colorectal carcinoma. For the *in vitro* cell experiment, GFP-tagged Rab11 plasmid was transfected into HT29 colon cells, E-cadherin expression and cell transformation were monitored by Western blot and confocal microscopy.

**Results:**

In immunohistochemistry, the mean score of E-cadherin in tumor and normal tissues was 1.41 ± 0.06 and 1.08 ± 0.06 (*p* < 0.05). The mean score of Rab11 in tumor and normal tissues was 0.51 ± 0.05 and 0.18 ± 0.02 (*p* < 0.05). Synchronous overexpression of E-cadherin and Rab11 was noted in 74 patients (66.5%) with colorectal carcinoma. When GFP-tagged Rab11 plasmid was overexpressed in cultured colon cell line HT-29, the E-cadherin expression was up-regulated, and cell membrane protrusion was induced, which resulted in cell transformation and cell migration.

**Conclusions:**

This study demonstrated the importance of the overexpression of Rab11 and E-cadherin in colorectal cancer. The results indicated that Rab11 together with E-cadherin might be potential markers for colorectal cancer progression and treatment.

## Background

Most tumors are epithelial based cell types. Epithelial Mesenchymal Transition (EMT) is thought to be a marker of tumor progression and metastasis. Normal epithelial cells express cadherin, catenin and other junctional adhesion proteins in the areas of cell-cell contacts; however, tumor cells that express mesenchymal markers have a greater tendency to be invasive and metastasize [1].

E-cadhrin has been considered to be a “tumor suppressor” marker, as the breakdown of cell-cell contacts promotes cell transformation and further migration. However, recent evidence demonstrated a promoting role of high expression of E-cadherin in aspects of tumor progression. An unexpected high expression of E-cadherin in tumor progression was observed in aggressive brain tumor [2] and in inflammatory breast carcinoma; E-cadherin was identified as being involved in the pathogenesis of advanced breast carcinoma [3, 4]. It has also been demonstrated in clinical studies that the E-cadherin and β-catenin mRNA levels were increased in colon cancer progression and in liver metastasis [5]. E-cadherin protein expression and localization have also been found to be increased in primary colorectal tumors [6]. However, the related biological meaning and the underlying cellular mechanism are still under investigation.

As the process of metastasis involves transformation of epithelial cells between EMT and MET, the expression of E-cadherin is regulated dynamically and does not just act in the role of tumor suppressor [7]. Recent reports have also pointed towards an alternative role of E-cadherin in carcinogenesis, which suggests that it may not just be that of a “sticky” molecular complex in between cells – the dysregulated over-expression of E-cadherin may participate in tumor progression through its associated cellular mechanisms [8–10]. In epithelial cells, cadherins and catenins form strong cell-cell contacts and are also dependent on vesicle-mediated intracellular transport. Continual trafficking of E-cadherin to form the cell junction is essential for morphogenesis [11, 12]. Increases of E-cadherin endocytosis and recycling have been shown to be correlated with cancer progression [13, 14].

Vesicles transport mediated through the endocytic system including endocytosis and recycling is controlled primarily by small GTPases of the Rab family [15]. Different Rab proteins are localized in cellular compartments and regulate distinct vesicles and endosome transport routes. It has been demonstrated that Rab proteins were associated with cancer metastasis [16–18]. Rab11 has been shown to function in recycling endosome movement to the membrane and regulate epithelial cell polarity [19, 20], and also been demonstrated to be related to hypoxia-stimulated cell invasion in breast carcinoma [21]. Hence, dysregulation of the expressions of Rab proteins may be an important component of human carcinogenesis, and a recent study also illustrated that Rab11-mediated recycling endosome is required for E-cadherin trafficking during epithelial morphogenesis. Active Rab11 can carry E-cadherin to the cell-cell contacts; however, the Rab11 inactive form fails to regulate recycling endosome for E-cadherin membrane targeting [22]. Although *in vitro* studies have demonstrated that Rab11 can regulate E-cadherin membrane targeting, its role in cancer cell transformation is still not clear, and the relationship with the tumor suppressor role of E-cadherin is still controversial.

Colorectal cancer is one of the major causes of death worldwide, and the E-cadherin expression dynamics may be critical in colorectal tumor progression. Thus, we speculated that Rab11-mediated E-cadherin turnover is an important mechanism in colorectal tumor formation. In this study, the expressions of E-cadherin and Rab11 were examined pathologically in colorectal tumor specimens, and Rab11 was also over-expressed in cultured colon cells for *in vitro* transformation study.

## Methods

### Patients and ethics statements

The study group consisted of 113 consecutive patients (age range, 24–93 years old, median age, 59 years old, 65 male, 48 female) who had undergone resection for localized colorectal cancer from April 1997 to December 2003 at Ching-Cheng General Hospital, Taiwan. The protocol was reviewed and approved by the Ching-Cheng General Hospital Institutional Review Board (HT110018). Written informed consent was obtained from all patients. Archival paraffin-embedded samples were used to build up tissue microarray blocks in the Department of Medical Technology of Yuanpei University in 2008. Patients with inflammatory disease, infection, bowel obstruction or perforation were excluded. Tumors were located in the ascending colon in 21 patients (19%), transverse colon in 6 patients (5%), descending colon in 5 patients (4%), sigmoid colon in 26 patients (23%) and rectum in 55 patients (49%). All primary cancerous tissues were excised.

Under TNM (AJCC, 7^th^ ed.) classification, 11 patients had stage I disease, 42 patients had stage II disease, 52 patients had stage III disease and 8 patients had stage IV disease. Colorectal carcinoma specimens and uninvolved mucosa specimens were obtained during surgery. All protein expression assessments for this study were carried out without knowledge of the pathological data.

### Cell culture and transfection

HT-29 and SW 480 colon cells (ATCC, VA, USA) were grown in Dulbecco’s modified Eagle’s Medium (DMEM) supplemented with 10% calf serum, penicillin and streptomycin (GIBCO-BRL, Gaithersberg, MD, USA) and kept in an incubator under 5% CO_2_ at 37°C. For transfection, cells were grown on 24-well plates in normal growth medium without antibiotics, and Lipofectamine 2000 transfection reagent (Invitrogen, CA, USA) was used for GFP-tagged Rab11 wild-type, dominant negative (DN) mutant (Addgene, MA, USA) and Rab11 shRNA plasmid (RANi core, Academia Sinica, Taiwan) transfection. Cells were analyzed 24 hr post-transfection, and the efficacy of transfection was confirmed by immunoblot analysis of cell lysates using a rabbit anti-GFP antibody (abcam, MA, USA).

### Immunohistochemistry

The tissue specimens were first fixed in 4% paraformaldehyde for 2 hrs. After dehydration, specimens were then embedded in paraffin blocks. 5-μm-thick paraffin sections were cut and deparaffinized in xylene substitute and rehydrated in graded alcohols and distilled water. Antigen retrieval was achieved by heating the samples without boiling in 0.01 M citrate buffer, pH 6.0, with 0.1% tween 20. This treatment was conducted twice for 10 min. The sections were washed in double distilled water (ddH_2_O). The endogenous peroxide was blocked by 0.3% hydrogen peroxide in methanol for 10 min. The sections were then incubated with E-cadherin (1:150) (BD Biosciences, USA) or Rab11 (1:80) antibodies (Cell signaling technology, MA, USA) at room temperature for 1 hr. A histostain-SP DAB kit (Invitrogen) was then used to reveal the primary antibody; the secondary antibody (reagent 1B in DAB kit) was incubated with the sections for 10 min. After washing in ddH_2_O thrice for 2 min, the sections were then incubated with streptavidin-peroxidase conjugate (reagent 2 in DAB kit) for 10 min. After washing, the final staining was performed in diaminobenzidine tetrahydrochloride (DAB) solution (reagent 3A-3C in 1 ml ddH_2_O) for 5 min. The nuclei were counterstained with Mayer’s hematoxylin (reagent 4 in DAB kit) for 3 min. After washing with ddH_2_O, the slides were then transferred through an ascending ethanol series (95%, 100%) and xylene substitute before mounting. The scoring used for immunohistochemistry was the “I” index [6], the equation for which is I = 0*f0 + 1*f1 + 2*f2 + 3*f3, where f0-f3 are the fractions of the cells showing a defined level of staining intensity (from 0–3); the numbers 0–3 represent the following: “0” negative, no detectable staining, “1” weak, but still detectable staining, “2” moderate, clearly positive but still weak; and “3” heavy and intense staining.

### Western blots

Tissue samples were cut into 2-3-mm pieces and homogenized in lysis buffer (1% NP-40, 50 mM Tris pH 7.4, 150 mM NaCl, 2 mM MgCl_2_, 1 mM EGTA, and protease and phosphatase inhibitors) using a homogenizer on an ice tray, and the protein concentration was determined by BCA reagent. Protein samples were mixed with sample buffer, boiled for 5 min and separated by SDS–PAGE. Proteins on gel were then transferred onto PVDF membrane, blocked in blocking buffer containing 5% BSA, and then probed with primary antibodies against E-cadherin, Rab11, vimentin ( Epitomics, CA, USA) or GAPDH (Santa Cruz, CA, USA ), followed by incubation with appropriate HRP-conjugated secondary antibodies. Blots were developed using an enhanced chemiluminescence system.

### Trans-well cell migration assay

HT-29 cells were transfected with GFP-Rab11 or Rab11 shRNA. After 48 hours, cells were trypsinized into trans-well insert (BD Biosciences) for cell migration assay. Transfected cells were transferred to the upper chamber of the trans-well insert that with 8 μm pore size and containing serum-free medium. Cells were allowed to migrate for 12 hours toward the bottom chamber which was filled with normal serum medium. Cells remaining on the upper membrane were removed by cotton swab. The migrated cells on the bottom side were fixed and stained with DAPI nuclear dye. The migrated cells were then revealed by fluorescence microscope and counted for quantification.

### Immunofluorescence microscopy

Cells grown on glass coverslips were fixed with 3.7% formaldehyde and permeabilized in 0.1% Triton-X 100. For the transfection experiment, cells were first grown on cover slips for 24 hrs and then transfected with GFP-tagged Rab11 wild-type or dominant negative plasmid for an additional 24 hrs. The fixed cells were incubated with mouse anti-E-cadherin antibody (1:100 dilution in PBS/0.1% Triton-100/3% BSA) at room temperature for 1 hr and then incubated with Cy3 conjugated anti-mouse secondary antibodies (1:200 dilution in PBS/0.1% Triton-100/3% BSA) at room temperature for 1 hr. Coverslips were mounted with Gel Mount aqueous mounting medium (Sigma, St. Louis, MO, USA). Images were acquired using a Zeiss LSM 510 META confocal microscope with a 63× objective (1.4 oil). To analyze the cell morphology changes for transformation, cells were scanned by a laser confocal microscope with z-sections for 3D image construction of the side view. The transfected cells chosen for scanning were either localized inside the cell colony or on the margin of the island.

### Statistics

Results are expressed as mean ± standard deviation. Chi-squared tests were used to compare categorical variables. The Student *t*-test was used to compare continuous variables. Differences at the *p* <0.05 level were considered statistically significant. For cell culture experiments, at least three independent experiments were performed.

## Results

### Elevated E-cadherin and Rab11 expressions were revealed in colon tumor tissues

This study first examined the E-cadherin and Rab11 expression patterns in nine colon carcinoma patients. Tumor tissue and non-tumor tissue including the distal and proximal ends of colon tissues from the same patient were collected. Tissues were processed for immunohistochemistry (IHC) with antibodies against E-cadherin and Rab11.

**Figure 1:**
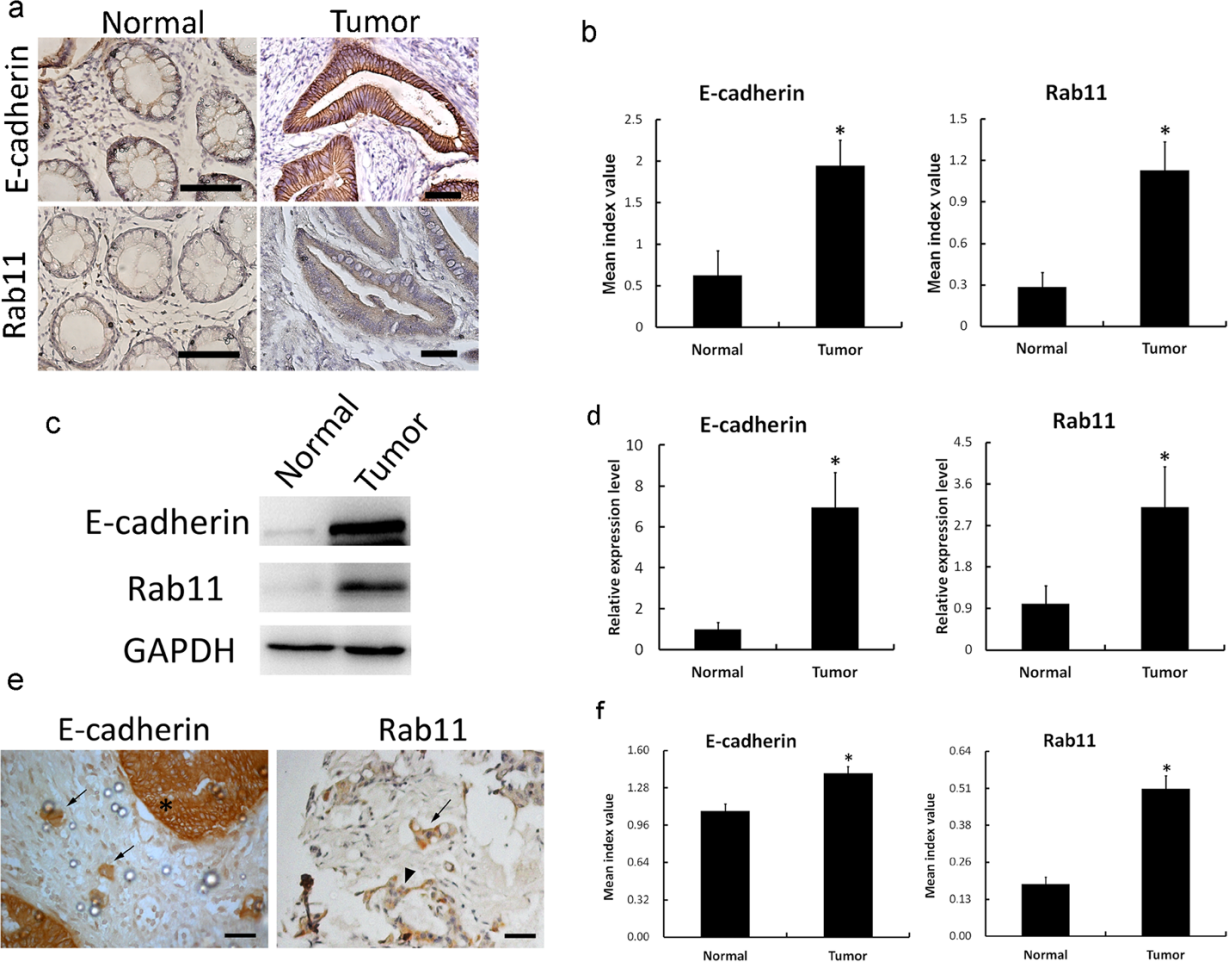
**E-cadherin and Rab11 expressions in colon tumor tissues. (a)** Colon tumor tissue and non-tumor (indicated to be normal) tissue samples from the same patient were processed for immunohistochemistry with antibodies against E-cadherin or Rab11; micrographs were taken using a Zeiss light microscope. Scale bar = 50 μm. **(b)** E-cadherin and Rab11 expressions in tissues from 9 patients were quantified by “I” index scoring as described in Methods. **(c)** Protein extracts from colon tumor tissue and non-tumor tissue were subjected to SDS-PAGE and Western blot analysis to determine the expression levels of E-cadherin and Rab11. GAPDH was used as the internal loading control. **(d)** Quantification of E-cadherin and Rab11 protein expressions in 9 patients by Western blot assay. Value = mean ± SD, * *p* < 0.05. **(e)** Immunohistochemistry study of colon tumor tissue showed the E-cadherin and Rab11 expressions in colon tumor infiltrated cells. The asterisk indicates the tumor region, arrows indicate the tumor infiltrated cell nest, and the arrowhead indicates a disarranged gland-like structure. Scale bar = 50 μm. **(f)** Colorectal tumor and non-tumor (indicated to be normal) tissue paraffin sections from 113 patients were mounted on glass slides as a tissue microarray. E-cadherin and Rab11 were detected by immunohistochemistry. The expressions of E-cadherin and Rab11 were scored and quantified by the “I” index as described in Methods. Value = mean ± SD, * *p* < 0.05.

In the IHC results, the mucosa cells of non-tumor tissue, which was indicated to be a normal tissue, exhibited the basal level expression of E-cadherin in most of the cell membrane; weak expression of Rab11 was detected in the epithelial cytoplasm. In colon tumor tissue, serious proliferation of the cancer cells was observed, and E-cadherin was found to be intensively expressed in the cell membrane; the Rab11 expression was also found to be increased in cancerous cells (Figure 1a). The intensities of E-cadherin and Rab11 expressions in tissues were scored and quantified using the “I” index as described in Methods, and the results showed that the expressions of E-cadherin and Rab11 in tumor tissues were significantly higher than in normal tissues in the preliminary 9 cases (Figure 1b). Tissue proteins were also extracted for Western blot analysis of E-cadherin and Rab11 expressions. Samples of one of the patients, demonstrated in Figure 1c, showed that E-cadherin and Rab11 were both highly expressed in tumor tissue. By Western blot analysis, the expression levels of E-cadherin and Rab11 were quantified in the initial 9 patients (Figure 1d). By immunocytochemistry, infiltrated tumor nests were also found in the stroma of colon tumor tissue, and E-cadherin and Rab11 were stained intensively in these infiltrated tumor cells (Figure 1e). These data suggested that E-cadherin and Rab11 might play important roles in colon tumor cell transformation and migration based on pathological evidence. In order to examine the relationship between E-cadherin and Rab11 expressions in colorectal tumors with statistical significance, tissue array chips were created from 113 patients and IHC staining with E-cadherin and Rab11 antibodies was performed. The quantified results showed that the expressions of E-cadherin and Rab11 (Figure 1f) were both higher in tumor parts than in non-tumor parts. The mean scores of E-cadherin in the tumor and normal mucosa were 1.41 ± 0.06 and 1.08 ± 0.06, respectively, which were significantly different (*p* < 0.05). In addition, the mean scores of Rab11 in the tumor and normal mucosa were 0.51 ± 0.05 and 0.18 ± 0.02, which were also significantly different between the two groups (*p* < 0.05).

In 84 patients (74.3%), the E-cadherin expression was higher in tumor tissues than in non-tumor tissues. The specimens of stage IV disease had the largest percentage of over-expression (87.5% versus 12.5%). The Rab11 expression was higher in tumor tissues than in non-tumor tissues in 100 cases (88.5%), especially specimens of stage III tumors, which showed the largest percentage of over-expression of Rab11 (94.2% versus 5.8%).

When E-cadherin and Rab11 were combined together for observation, over-expression of both proteins was presented in 74 patients (65.5%). Solitary over-expression of Rab11 was found in 26 patients (23.0%) and solitary over-expression of E-cadherin was found in 10 patients (8.9%). Only 3 patients (2.7%) lacked any over-expression of the two proteins. Furthermore, all of these patients were classed as stage I or II. However, there was no significant difference between the stages with different expressions of E-cadherin or Rab11 (p = 0.09). These results suggested that the elevation of E-cadherin or Rab11 plays an important role in colorectal carcinoma formation regardless of stage (Table 1).

**Table 1:** **E-cadherin and Rab11 expression patterns in 113 colorectal carcinoma patients**

No. of cases, (%)				
	E-cadherin + Rab11+	E-cadherin - Rab11 +	E-cadherin + Rab11-	E-cadherin - Rab11 -
Stage I	9/11(81.82%)	1/11(9.09%)	0/11(0%)	1/11(9.09%)
Stage II	23/42 (54.76%)	13/42 (30.95%)	4/42 (9.52%)	2/42 (4.76%)
Stage III	38/52 (73.08%)	11/52 (21.15%)	3/52 (5.77%)	0/52 (0%)
Stage IV	4/8 (50.00%)	1/8 (12.50%)	3/8 (37.50%)	0/8 (0%)
Total patients	74/113 (65.49%)	26/113 (23.01%)	10/113 (8.85%)	3/113 (2.65%)

The symbols (+) and (-) indicate the expression level according to tissue scoring in the tumor part being higher or lower as compared with normal tissue.

### Proportional expressions of E-cadherin and Rab11 proteins were associated with epithelium morphology in cultured HT-29 colon cells

**Figure 2:**
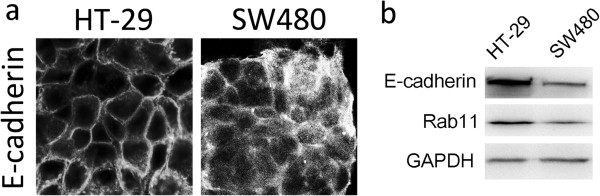
**Rab11 and E-cadherin expressions in cultured colon cells. (a)** HT-29 and SW480 colon cells were grown on coverslips, cells were fixed and stained with E-cadherin antibody, and E-cadherin was then revealed by Cy2-conjugated secondary antibody and observed by confocal microscopy. **(b)** HT-29 and SW480 cell lysates were collected and subjected to SDS-page. E-cadherin and Rab11 were detected by Western blot, and GAPDH was used as the internal loading control.

To examine the functions of E-cadherin and Rab11 *in vitro*, colon cell line HT-29 was used in this study. HT-29 cells have been demonstrated to be an ideal cell model for cell differentiation [23]. The phenotype of HT-29 was first examined and compared with another colon cell line, SW480. Cells grown on coverslips were fixed, and the E-cadherin expression was analyzed by immunofluorescent microscopy. An organized membrane E-cadherin staining pattern was revealed in HT-29 cells; however, the distribution of E-cadherin was shown to be diffused in SW480 cells (Figure 2a). The Western blot results showed greater Rab11 and E-cadherin protein expressions in HT-29 cells than in SW480 cells (Figure 2b). The results suggested that the proportional expression levels of junctional adhesion protein E-cadherin and recycling endosome protein Rab11 are associated with colon cell morphology.

### Rab11 overexpression in colon cells up-regulated E-cadherin expression and induced membrane protrusion and cell migration

**Figure 3:**
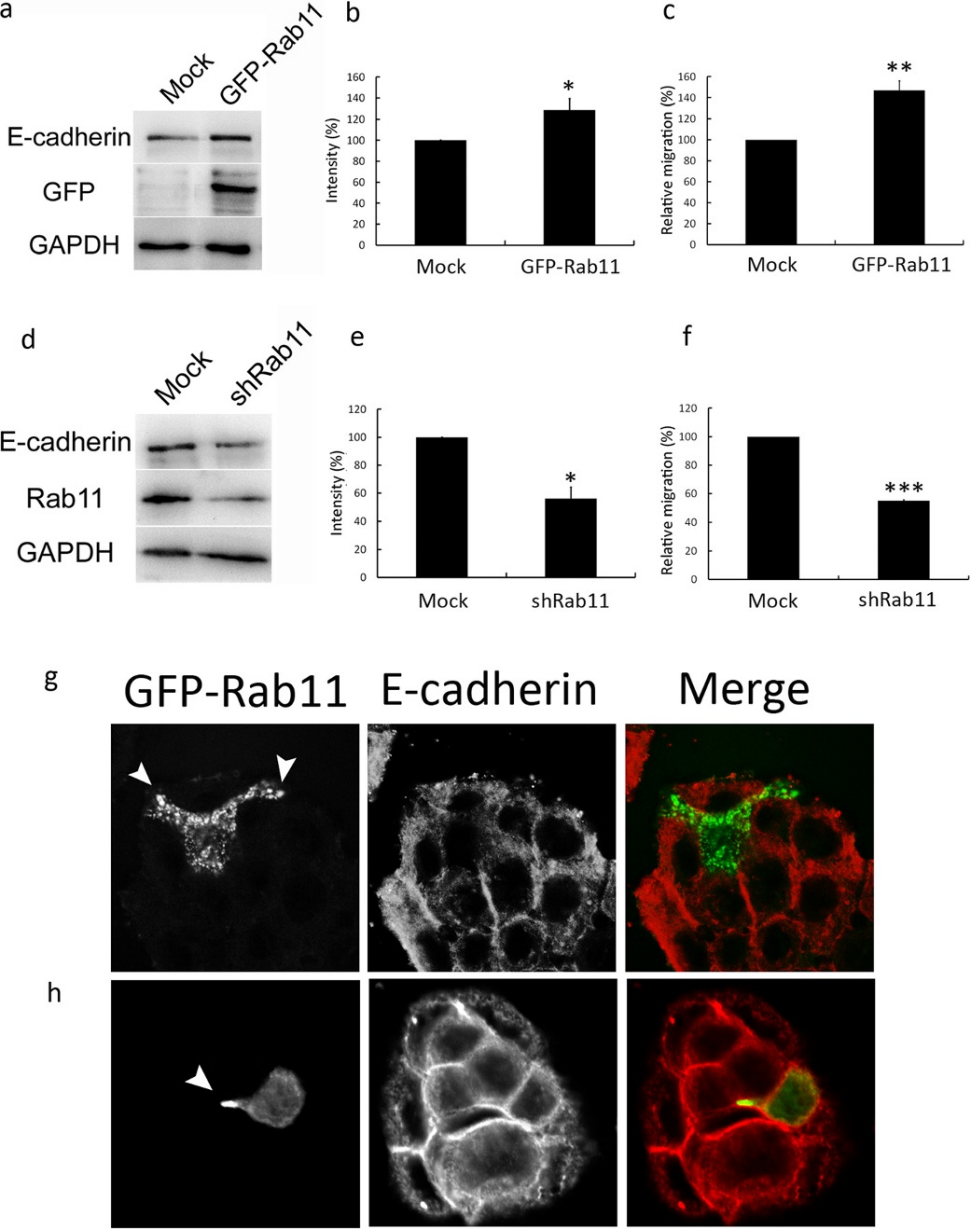
**Rab11 overexpression induced E-cadherin expression and cell migration in HT-29 cells.** HT-29 cells were transfected with GFP-tagged Rab11 plasmid **(a-c)** or plasmid constructed with Rab11 shRNA **(d-f)**. After 24 hr, cell lysates were collected and subjected to Western blot analysis for GFP-Rab11, E-cadherin and Rab11 expressions using anti-GFP, anti-E-cadherin and anti-Rab11 antibodies, respectively. GAPDH was used as the internal loading control. Quantifications of E-cadherin expression in GFP-Rab11 overexpressed cells (b) and in Rab11 knockdown cells **(e)** are shown from at least three independent experiments. Value = mean ± SD, * *p* < 0.05. Trans-well migration assay for Rab11 overexpression **(c)** and knockdown cells **(f)** were quantified. Value = mean ± SD were from three independent experiments, ** *p* < 0.01; *** *p* < 0.001. HT-29 cells grown on glass coverslips were transfected with GFP-Rab11; cells were then fixed and stained with anti-E-cadherin antibody, followed by staining with Cy3 conjugated secondary antibody. **(g)** GFP-Rab11-overexpressing cell migration out of the cell colony or **(h)** migration towards the center of the cell colony was observed by confocal microscopy. Arrowheads indicate cell membrane protrusion.

In order to determine the effects of Rab11 in colon cell morphogenesis, GFP-tagged Rab11 plasmid was overexpressed in HT-29 colon cells. Western blot results showed that the E-cadherin expression was up-regulated in Rab11-overexpressing cells (Figure 3a, b). Rab11 overexpression also promoted cell migration significantly (Figure 3c, *p* < 0.01). When cells were treated with shRNA that targets Rab11, the depletion of Rab11 expression suppressed E-cadherin expression (3d, 3e), and inhibited cell migration (Figure 3f, *p* < 0.001). HT-29 cells transfected with GFP-tagged wild-type Rab11 plasmid were stained with E-cadherin antibody and analyzed by immunofluorescent microscopy. The results showed that GFP-Rab11 was distributed in the cell leading edge and had induced the cell membrane protrusion. When the Rab11-overexpressing cell was localized at the margins of the cell colony and started to migrate out of the colony, the expression of E-cadherin in the cell membrane was shown to be decreased (Figure 3g). When the GFP-Rab11-overexpressing cell was migrating towards the center of the cell colony, the transfected cells still showed Rab11-induced membrane protrusion; however, E-cadherin was stained intensively in the cell-cell contacts (Figure 3h). These results demonstrated that the overexpression of Rab11 induced HT-29 membrane protrusion and resulted in two distinct E-cadherin expression patterns depending on the location of the cells: E-cadherin was down-regulated when the cell was migrating out of the cell colony, but E-cadherin was strengthened on the cell membrane when the cell was migrating in the cell colony between neighbor cells.

### Rab11 induced colon cell transformation

**Figure 4:**
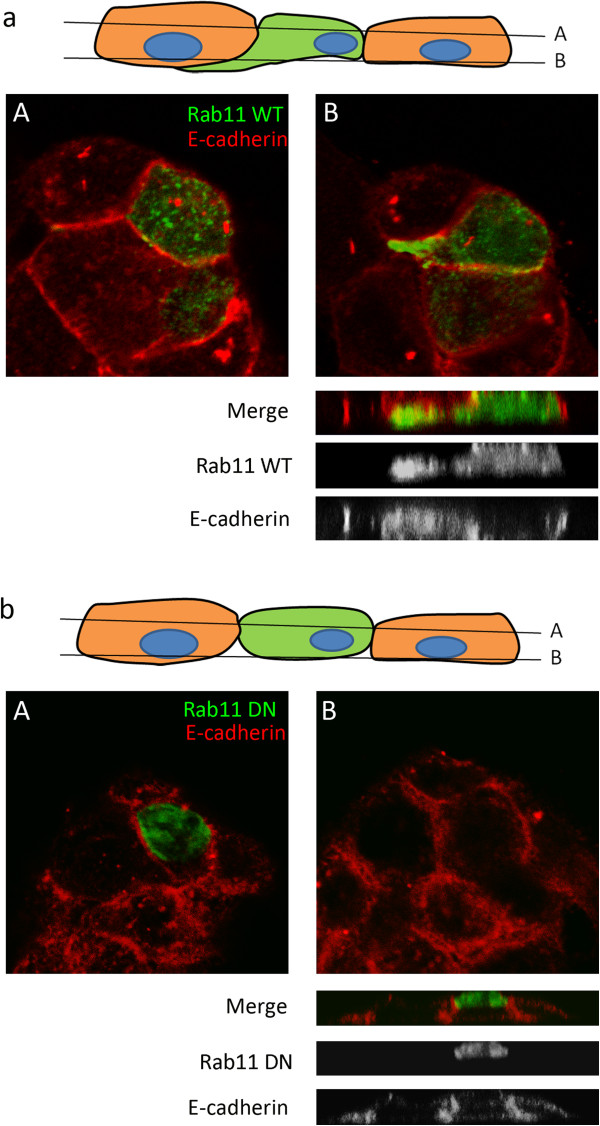
**Rab11 overexpression promoted cell transformation. (a)** GFP-Rab11 wild type and **(b)** GFP-Rab11 dominant negative over-expressing HT-29 cells were stained with E-cadherin antibody and analyzed by a confocal microscope as described in Figure 3h. The Z-section scanning images in positions A and B (as indicated in the diagram above) are shown, and Z-stacked images were composed for a 3D side view.

HT-29 cells transfected with Rab11 as shown in Figure 3f were further analyzed by a confocal microscope with z-sections. A 3D image was composed from the z-section images. The side view image showed that the Rab11 overexpressed cells (Figure 4a) induced cell transformation, resulting in cell migration; the Rab11 overexpressed cells migrated beneath a neighbor cell (see 3D side view). However, when cells were transfected with GFP-tagged dominant negative (DN) Rab11, the GFP-Rab11 DN was evenly distributed in the cytosol and did not induce cell transformation and migration (Figure 4b). This result indicated that overexpression of Rab11 induced HT-29 cell transformation, which was dependent on Rab11 activity.

## Discussion

E-cadherin, which functions as an adhesion junction, has been demonstrated to be an important marker in cancer biology, as disassembly of E-cadherin is required for epithelial cell transformation and migration. E-cadherin is bound with most of the β-catenin located at the cytosolic side and also interacts with the actin cytoskeleton. β-catenin is the key component of the Wnt pathway that can be stimulated for cell growth and proliferation [10]. E-cadherin has also been shown to regulate initial cell-cell contacts formation and can further recruit exocyst components to the contacts for the formation of cell surface polarity [24]. Recently, the dark side of E-cadherin has been revealed gradually in pathological study of different cancers. The increased expression of E-cadherin was found to be associated with the formation of epithelium tumors. E-cadherin may play an important role in “collective cell migration” [25] and provide the “anchorage-independent” property of cancer cells [9]. In our study, significant over-expression of E-cadherin was found in colorectal cancer tissues. These findings are consistent with the previous study of Truant et al. [5], who demonstrated that the expression ratio of E-cadherin in reference to the normal adjacent tissue was increased in 57% of primary tumors. In contrast, only 30% of specimens were decreased in terms of the expression of E-cadherin in colorectal carcinoma. Regarding liver metastasis, a significantly higher expression of E-cadherin was found at stage I ~ II than at stage III ~ IV [6]. Therefore, the authors of the study suggested that the role of high expression of E-cadherin in colorectal cancer cells may be protective against widespread metastasis.

As Rab11, which functions as a recycling endosome, has been reported to play a role in regulating E-cadherin turnover *in vitro*, dysregulation may be associated with cancer formation. Our data subsequently demonstrated that the expression of Rab11 was also increased in colorectal cancer tissues. Co-overexpression of E-cadherin and Rab11 was found in 65.5% (74/113) of colorectal carcinomas, which suggests that dysregulation of both molecules might be associated with the occurrence and progression of colorectal carcinoma. The correlations of expressions of E-cadherin and Rab11 with stages of colorectal carcinoma were not statistically significant. However, the number of E-cadherin and Rab11 co-expressing cases was decreased in advanced stage cancers (81.8% in stage I, but 50% in stage IV). As we know, pathological progression from early adenomatous proliferation through adenomatous polyp, high grade dysplasia and ultimately, invasive colorectal carcinoma to metastasis occurs as a continuum. This progression coincides with the accumulation of multiple genetic alterations during neoplastic progression as originally described by Fearon and Volgelstein [26]. The regulatory alteration of E-cadherin and Rab11 may vary dynamically in the multistep model of progression of colorectal carcinoma in different stages.

E-cadherin may participate in tumor progression through its associated cellular mechanisms. In epithelial cells, β-catenin acts as a linker between transmembrane E-cadherin and cytosolic actin fibers and forms a junctional adhesion complex. β-catenin is either stable connected to E-cadherin or translocated into the nucleus and binds to Lef/Tcf transcription factor upon stimulation for cell proliferation. Free cytosol β-catenin is degraded through ubiquitin-mediated degradation. Therefore, dynamic regulation of membrane E-cadherin and Rab11 may be necessary for cell proliferation and tumor growth. Rab11 was suggested to play a role in E-cadherin recycling and enhance membrane E-cadherin dynamics, which may be involved in cell signaling for cancer cell growth.

The *in vitro* experiments used GFP-Rab11 plasmid overexpressed in HT-29 cells induced cell transformation and migration. Intense staining of E-cadherin and Rab11 were also observed in infiltrated tumor nest cells. Actin dynamics are required for cell motility, and it has also been demonstrated that Rab11 interacts with RTK down-stream target Rac1 and controls moesin activity to regulate the endocytic cycle and actin cytoskeleton in cell migration and collective cell migration [27–29]. Rac1 has been shown to regulate actin nucleation via neural WASP (N-WASP) and the down-stream Arp2/3 complex [30]. A recent study has also shown that Rac1 activation is involved in twist1-induced cell transformation and migration [31]. Taken together, Rab11 plays a role not just in E-cadherin turnover but also improves the cytoskeleton reorganization for cell migration.

Discovering markers for cancer progression is important; however, the formation of cancer is multi-stepped, and specific protein expressions are restricted and responsible for different steps of EMT. Cell migration is an integrated process that requires continuous, coordinated formation and disassembly of adhesions. These processes are complex and require regulated interaction of numerous transcription factors such as snail/slug or twist, and activation of specific signaling pathways [32–35]. In this study, the expression of Rab11 was shown to be associated with cancer formation, and the expression of Rab11 also induced cell transformation, which is associated with cell motility. Moreover, Rab 11 could upregulate the expression of E-cadherin. Thus, Rab11 may be a useful maker together with E-cadherin for the diagnosis of colorectal cancer progression. However, the roles of other transcription factors in this mechanism have not been elucidated and require further investigation.

In the report of Anastasiadis et al. [9], several possible mechanisms were suggested to explain the positive role of E-cadherin in tumor progression. First, E-cadherin may cross-talk with EGFR signaling. Second, increased E-cadherin expression may up-regulate the expression of anti-apoptotic proteins, such as Bcl-2 and Bcl-xL. Third, E-cadherin may trigger the activation of the PI3K/AKT pathway through p85. In addition, extracellular N-terminal E-cadherin ectodomain “shedding” could be a role in tumor-promoting activities [9]. Our results showing that Rab11 up-regulated E-cadherin to induce the transformation of cancer cells might be another mechanism of alteration of neoplastic progression by E-cadherin.

## Conclusions

Our data for the first time demonstrated that Rab11 regulated E-cadherin expression and promoted colon cancer cell transformation. Overexpression of both E-cadherin and Rab11 was also detected in the majority of colorectal carcinoma samples. Regulation of the dual protein motifs might facilitate targeting of the progression of colorectal carcinoma in the future.
